# A Neonatal Nurse‐Controlled Model of Analgesia to Manage Post‐Operative Pain in the Surgical Neonate: A Pilot Randomised Controlled Trial

**DOI:** 10.1111/jan.16992

**Published:** 2025-04-24

**Authors:** Renee Muirhead, Kathryn Kynoch, Glenda Hawley, Emma Ballard, Pita Birch, P. A. Lewis

**Affiliations:** ^1^ Neonatal Critical Care Unit Mater Health South Brisbane Queensland Australia; ^2^ School of Nursing, Midwifery and Social Work, University of Queensland Brisbane Queensland Australia; ^3^ Mater Health And Queensland Centre for Evidence Based Nursing and Midwifery: A JBI Centre of Excellence Newstead Queensland Australia; ^4^ School of Nursing, Queensland University of Technology Kelvin Grove Queensland Australia; ^5^ QIMR Berghofer Medical Research Institute Brisbane Queensland Australia

**Keywords:** analgesia, feasibility, neonatal, nurse‐controlled, pain, pilot, post‐operative, surgical

## Abstract

**Aim:**

To test the feasibility and acceptability of a newly developed model of neonatal nurse‐controlled analgesia to manage pain in the post‐operative infant.

**Design:**

The study utilised a single‐centre two‐arm parallel, unblinded randomised controlled external pilot trial design.

**Methods:**

The pilot trial was conducted in a surgical neonatal tertiary intensive care unit in Brisbane, Australia. Eligible infants were randomised to receive either post‐operative pain management care via a model of neonatal nurse‐controlled analgesia or standard care. Feasibility and acceptability were the primary outcomes. Seven feasibility outcomes were assessed by a traffic light system to delineate progression to a larger trial. Acceptability and clinical utility of the model of care by staff were assessed by feedback from an anonymous questionnaire that was administered at the completion of the trial period. Secondary outcomes included parental attitudes and perceptions of post‐operative pain management to help establish primary outcomes for a larger randomised controlled trial.

**Results:**

Overall staff found the formalised model beneficial for managing post‐operative pain but found the complexity of the model and ability to titrate analgesia based only on documented pain scores barriers requiring further consideration. Three of the seven feasibility outcomes failed to reach ‘greenlight’ targets to progress to a larger trial with adherence to the model, and the proportion of eligible infants not recruited was allocated a ‘redlight’. Secondary outcomes were comparable and support future study.

**Conclusion:**

This pilot feasibility study has shown that a model of neonatal nurse‐controlled analgesia can be safely implemented and utilised in the post‐operative care of the surgical neonate. Further exploration of the barriers to model adherence and recruitment is warranted before a future larger trial is undertaken.

**Impact:**

Though not all primary outcomes reached an acceptable range for further progression, this pilot feasibility study provided invaluable learning and has provided direction for future research into the provision of a family integrated and responsive model of analgesia.

**Reporting Method:**

This study is reported in line with the Consolidated Standards of Reporting Trials (CONSORT): Extension to randomised pilot and feasibility trial and the TIDieR Checklist (Template for Intervention, Description and Replication).

**Public or Patient Contribution:**

No patient or public contribution was utilised for this study.

**Trial Registration:** ACTRN12623000643673—the trial was prospectively registered

**Summary**

- What already is known?
  ○ Research has demonstrated that not only do newborn infants experience immediate physiological and hormonal responses to pain, but untreated or poorly managed pain is associated with alterations in brain development leading to adverse neurodevelopmental sequelae.
  ○ There is limited evidence on the optimal approach to managing neonatal post‐operative pain to alleviate discomfort, while minimising the unwanted effects of excessive use of analgesics.
  ○ The use of a validated neonatal pain assessment tool, pharmacological support and the implementation of parent or nurse‐delivered comfort interventions can help to reduce and manage pain and discomfort in the neonatal population.
- What this paper adds?
  ○ Parental involvement in post‐operative analgesic care is feasible but further research into how to engage families in the early post‐operative period is warranted.
  ○ That a model of neonatal nurse‐controlled analgesia can be used safely and effectively to manage neonatal post‐operative surgical pain and discomfort.
- Implications for practice/policy
  ○ This pilot revealed important directions for future research, including the exploration of new approaches to increase recruitment of study participants in the early newborn period and of the barriers to achieving overall compliance to the delivery of a model of neonatal nurse‐controlled analgesia.

## 1 Introduction

Up until the late 1980s, there was a prevailing belief among medical practitioners and health care clinicians that newborn infants did not experience pain (Kuratani 2015). Subsequently, surgical procedures were often performed without analgesia, and pre‐emptive analgesia was not provided prior to painful procedures in the neonatal intensive care unit (Kuratani 2015; Walker 2014). Since this time, extensive research has demonstrated that not only do newborn infants experience immediate physiological and hormonal responses to pain (Anand and Hickey 1987), but untreated or poorly managed pain was associated with alterations in long‐term pain processing ability and brain development leading to adverse neurodevelopmental sequalae (Walker 2013). As a result, opioid therapy has become the mainstay of post‐operative pain management due to its ability to diminish both the physiological and behavioural effects of pain and stress (Campbell‐Yeo et al. 2022).

Opioid therapy in the newborn infant has been shown to be associated with several adverse outcomes including: over‐sedation, prolonged mechanical ventilation, increased length of stay and the subsequent development of iatrogenic withdrawal syndrome (IWS), a syndrome of tolerance and dependence that has deleterious physiological effects on the vulnerable newborn (Best et al. 2015). Furthermore, animal and in vitro studies suggest neurotoxicity in relation to opioid usage in the newborn (Attarian et al. 2014; Bajic et al. 2013). These complications and adverse outcomes in infants, in addition to emerging evidence that the surgical newborn is at greater risk of poorer neurodevelopmental outcomes (Peters et al. 2005; Walker et al. 2015), indicate that an accurate assessment and a prompt consistent approach to the management of postoperative pain is warranted. A multi‐modal family integrated approach incorporating both pharmacological and non‐pharmacological pain‐relieving strategies may provide the neonatal clinician with a more targeted, responsive and individualised method of pain relief that alleviates discomfort while minimising the unwanted effects of excessive use of analgesics.

The use of non‐pharmacological measures such as non‐nutritive sucking, skin to skin contact, containment and parental presence has all been demonstrated to have pain relieving properties (Shen et al. 2022). Concurrent use of these measures with opioid therapy in infants admitted to the intensive care unit has shown a reduction in infant pain scores and the amount of opioid administered (Bapat et al. 2023). The use of these comfort measures by family members not only increases opportunities for shared decision making and participation in the management of their infant's post‐operative pain (Jyoti et al. 2023; Ullsten et al. 2021) but has a positive impact on both parental and neonatal outcomes (Ullsten et al. 2021).

A model of neonatal nurse‐controlled analgesia (NNCA) utilising a family integrated approach and incorporating both pharmacological and non‐pharmacological strategies to optimise post‐operative pain management was previously developed under the guidance of an e‐Delphi study (Muirhead et al. 2024). The primary aim of this prospective pilot study was to test the feasibility and acceptability of this newly developed NNCA pain management model compared to standard clinical care in infants of 35 or more weeks of gestation.

## 2 Methods

This single‐centre two‐arm parallel, unblinded randomised controlled external pilot trial was reported following the Consolidated Standards of Reporting Trials (CONSORT): Extension to randomised pilot and feasibility trials (Eldridge et al. 2016). The study was approved by the Human Research Ethics Committee (HREC) of the research hospital in 2023 (HREC/MML/94314) and was prospectively registered (ACTRN12623000643673) with the Australian New Zealand Clinical Trials Registry on June 16, 2023.

### 2.1 Setting

The study took place in the cardiac/surgical intensive care area of the Neonatal Critical Care Unit (NCCU) at a tertiary referral hospital in Brisbane, Australia. The cardiac/surgical intensive care unit has a 17‐cot capacity and provides care to over 200 surgical babies annually. Data were collected between June 2023 and May 2024.

### 2.2 Procedure

Potential participants were identified from both the daily list of planned births of infants known to have a surgical condition diagnosed antenatally and from the daily inpatient record. The inclusion criteria for study participation were: infants (1) admitted to the NCCU for neonatal surgery, (2) greater than or equal to 35 weeks post menstrual age, (3) opioid naïve or commenced opioids for the first time within the previous 48 h, (4) hemodynamically stable as determined by the treating medical team, (5) able to be cared for in a 1:1 nurse allocation for at least the first 24 h post‐operatively by a nursing clinician with a minimum of 2 years neonatal surgical experience and (6) parents being available to provide informed consent. Infants with any illness complicated by physical instability (e.g., Persistent Pulmonary Hypertension of the Newborn) in which additional sedation/muscle relaxation is required to manage the clinical condition were excluded.

### 2.3 Blinding and Randomisation

The study was an unblinded design where parents were approached by the research nurse following birth and at least 30 min prior to surgery and invited to participate in the trial. If informed consent was obtained, infants were randomised 1:1 to the current standard post‐operative pain management (control group) or the NNCA model (intervention group) using permuted block sizes of 2 and 4. The randomisation scheme was created for 26 participants by a statistician on the research team using STATA 17 (Stata Corp LLC, College Station, TX). Sequentially numbered opaque envelopes were used to conceal the randomised sequence from the research nurse until the intervention was assigned following parental consent.

### 2.4 Protocol

#### 2.4.1 Control

Infants allocated to the control group received current standard medical management for post‐operative pain. Standard medical care is prescribed at the discretion of the neonatologist/treating medical team. Morphine is generally the first‐line analgesic of choice for managing post‐operative pain in the NCCU; however, other analgesic and sedative agents such as fentanyl, paracetamol, dexmedetomidine and midazolam could be prescribed. Titration and weaning of analgesics are completed following review with medical team members. Pain scores, using the validated Modified Pain Assessment Tool (mPAT) (O'Sullivan et al. 2016), and a suggested opioid weaning guideline (Muirhead and Kynoch 2019) could be utilised to help guide the decision to titrate analgesia. The mPAT tool uses a combination of 10 behavioural and physiological responses with a possible total score of 20. The authors of the mPAT recommend scores > 5 should result in the use of non‐pharmacological comfort measures and scores > 10 should result in the use of analgesia (O'Sullivan et al. 2016). A detailed legend for non‐pharmacological measures is available on the standard mPAT scoring tool used in the NCCU, but there is no guide for their utilisation or recommendation for active parental involvement in managing post‐operative pain. mPAT assessment is recommended to be attended hourly for the first 24 h post‐operatively and then two to four hourly thereafter until time of discharge. Despite the availability of a weaning guideline and mPAT tool to assist the titration of analgesia, there is no directive or protocol/algorithm available to standardise overall analgesic management in the unit.

#### 2.4.2 Intervention

Infants in the intervention arm utilised the model of NNCA developed to manage post‐operative neonatal pain (Muirhead et al. 2024). Infants commenced using pathway A of the model (used for the first 12 h post‐operatively) on return from theatre. Depending on whether the infant had already commenced an opioid infusion prior to surgical intervention and/or the amount of analgesia administered intra‐operatively, consideration of a loading dose of morphine occurred on return to the neonatal unit following a medical review. Nursing staff were able to administer a bolus of 25 micrograms/kg of morphine every 15 min during the first hour post‐operatively to a maximum dosage of 75 micrograms/kg to achieve a mPAT score ≤ 4. If three boluses were needed, the opioid infusion rate was able to be increased by 5 micrograms/kg/h. Intravenous paracetamol 7.5 mg/kg was commenced within 2 h of commencing on Pathway A if not administered in the previous 6 h. Nursing staff were required to reassess infant pain scores in 60 mins. If the infant was achieving a mPAT score ≤ 4, the infant was able to commence on the *LOW* (green) pathway. If mPAT scores were between 5 and 9, the infant was to utilise the MOD (purple) pathway, and if mPAT scores were ≥ 10, the infant employed the *SEV* (blue) pathway. If after 12 h the infant's mPAT scores remained ≤ 9, transition to pathway B should occur. If mPAT scores were ≥ 10, the infant remained on pathway A until low pain scores were achieved or the NNCA paused due to escalation of pharmacological agents beyond accepted NNCA parameters. If the infant continued on analgesia at 5 days post‐operative, transition to pathway C was to occur.

In the intervention arm, the use of non‐pharmacological measures was considered first‐line analgesic therapy and parents were encouraged to be present and to deliver appropriate comfort interventions as clinically appropriate and in partnership with the primary care nurse. At the time of recruitment, families of the infants randomised to the treatment arm also received a brochure on strategies to help comfort their baby after surgery that was developed by the research team for this study.

All patients regardless of trial arm designated in the pilot study had continuous pulse and cardiovascular monitoring. Standard protocol requires hourly mPAT scores at a minimum for the first 24 h post‐operatively. Pain scoring assessment subsequent to this is 2–4 hourly, or more frequent if clinically indicated. Enrolled patients in the intervention arm, who at any time required medications or sedation to manage clinical hemostability, were to have the NNCA paused. If clinical condition improved and adjunctive sedatives or other medications ceased, the infant was able to recommence on the NNCA model. The NCCA model continued to be utilised until 48 h post cessation of opioid therapy. A copy of the NNCA model (Muirhead et al. 2024) can be found in Appendix S1.

### 2.5 Outcome Measures

Data collection began at enrolment and continued until the infant was either discharged home, transferred from the cardiac/surgical intensive care unit or required the recommencement of opioid therapy to manage postoperative pain subsequent to further surgical intervention. The Principal investigator was responsible for reviewing documented pain scores and subsequent adherence to the protocol algorithm for each 24 h period the NNCA was being utilised. For this pilot study, primary outcomes focused on feasibility and acceptability measures, while secondary outcomes measured effectiveness of the intervention.

#### 2.5.1 Primary Outcomes: Feasibility and Acceptability

**FIGURE 1:**
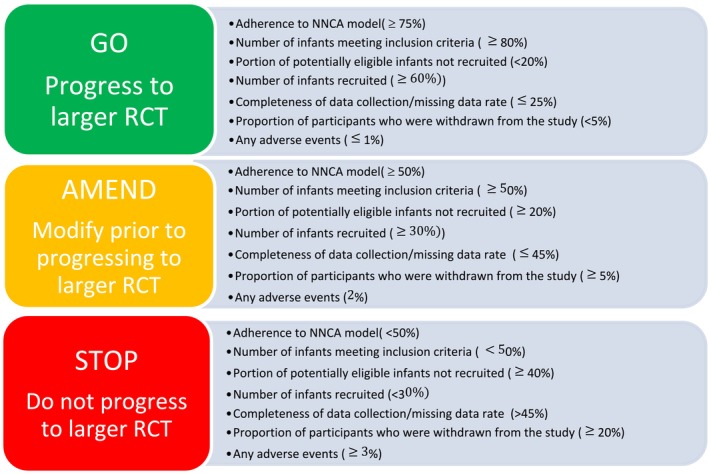
Feasibility progression criteria.

Feasibility of the study design was assessed by the research team and included data on recruitment processes, randomisation, data collection and retention. A traffic light system was utilised to determine progression criteria for a larger randomised control trial (RCT). The ‘traffic light’ red (do not proceed), amber (proceed with modifications) and green (proceed) system is a method of representing the progression for a trial in a simple and transparent way and has been used widely in other feasibility studies (Avery et al. 2017; Herbert et al. 2019). There is a lack of literature on acceptable progression criteria in neonatal analgesic‐related studies in the literature. Therefore, proposed percentages for this pilot study were determined following discussions with a neonatal stakeholder group, examining previous admission criteria of infants admitted to the surgical unit and reviewing recruitment and compliance data of studies conducted on infants that examined either protocolised opioid use or other analgesic strategies to manage neonatal pain (Curley et al. 2015; Czarnecki et al. 2020) (Figure 1).

Staff perception of acceptability and utility of the NNCA model in clinical care was determined by a six‐question survey developed by the research team using a 5‐point Likert scale from 1 strongly agree to 5 strongly disagree. Staff involved in caring for infants' receiving the intervention had the opportunity to complete this survey electronically via the Checkbox platform. Questions focused on use of the tool, impact on practice, perceived benefit, promotion of nurse autonomy and strengths and limitations. Staff were also provided the opportunity to provide qualitative feedback. Implied consent was obtained through staff voluntarily completing the survey. The link to the survey was emailed to all staff in the clinical area trialling the model at the end of the study period.

#### 2.5.2 Secondary Outcomes

Secondary outcomes are described in Table 1. Parents experience of pain management for their infant in the neonatal surgical unit was also sought from both the control and intervention group participant parents using the Parent Attitudes about Infant Nociception (PAIN) tool (Franck et al. 2004). This 40‐item self‐reported questionnaire aims to measure parents' attitudes about their infant's pain, including their expectations as parents (scale 0 to 10 with 0 = no pain to 10 = worst pain), involvement and participation in comfort care (6 point Likert scale where 1 = strongly agree to 5 = strongly disagree, two 4 point Likert scales where 1 = a lot to 4 = none and 1 = always to 4 = never) and satisfaction of pain management practices (6 point Likert scale where 1 = strongly agree to 6 = strongly disagree).

**TABLE 1:** Secondary outcomes results.

Secondary outcomes	Measures
Intensity of pain scores	mPAT pain scores will be measured retrospectively for each 24 h period. mPAT tool is a composite pain score out of a possible 20. Scores < 5 indicate nil/mild pain, scores 5–10 indicate mod pain and scores > 10 severe pain requiring pharmacological agents (O'Sullivan et al. 2016)
Overall opioid consumption	Overall opioid consumption measured in micrograms for each 24 h period
Time on invasive respiratory support	Time on invasive ventilation measured by number of hours participant was intubated
Time to reach full feeds	Number of hours until infant breast feeding on demand or tolerating full enteral feed quota as determined by treating medical team
Time to cessation of all analgesics	Number of hours for all analgesics to be ceased
Length of stay	Length of stay measured by number of days participant was admitted to the cardiac/surgical neonatal unit
Iatrogenic withdrawal	Iatrogenic withdrawal scores > 3 as measured by WAT‐1 tool. The WAT‐1 is a validated assessment instrument for monitoring opioid and benzodiazepine withdrawal symptoms. Scores > 3 may be indicative of iatrogenic withdrawal (Franck et al. 2008)
Management of Iatrogenic withdrawal	Pharmacological management of Iatrogenic withdrawal syndrome
Parent satisfaction with pain management	Parent attitudes about infant nociception (PAIN) tool (Franck et al. 2004)

### 2.6 Sample Size

This study was conducted to explore the feasibility of the intervention and not statistical significance; therefore, a power calculation was not performed. Allowing for attrition, an estimated sample size of 26 participants was determined, with 13 participants in each arm required to explore the feasibility outcomes of this study. For studies measuring feasibility aims and acceptability and not precision, participant rates of 10–15 per group are acceptable and have been used in other studies (Julious 2005; Thabane et al. 2010).

### 2.7 Statistical Analysis

Data was analysed using SPSS Statistics Version 29 (IBM Corporation, Armonk, NY, USA).

Categorical data were described using frequency and percentage and continuous data using mean and standard deviation for normally distributed data and median and interquartile range (IQR: 25th percentile and 75th percentile) for not normally distributed data. Ordered categorical data was reported as median (IQR: 25th percentile and 75th percentile). The comparison of interest was between NNCA and standard care, with the size of the difference between groups discussed where applicable. Likert responses to the staff survey were further collapsed into agree (strongly agree/agree) and disagree (strongly disagree/disagree) or similar to aid in interpretation based on the observed distribution of responses to each question. A modified intention‐to‐treat approach (mITT, all randomised patients with at least one evaluation pertaining to outcome of interest) was utilised for this pilot study.

## 3 Results

**FIGURE 2:**
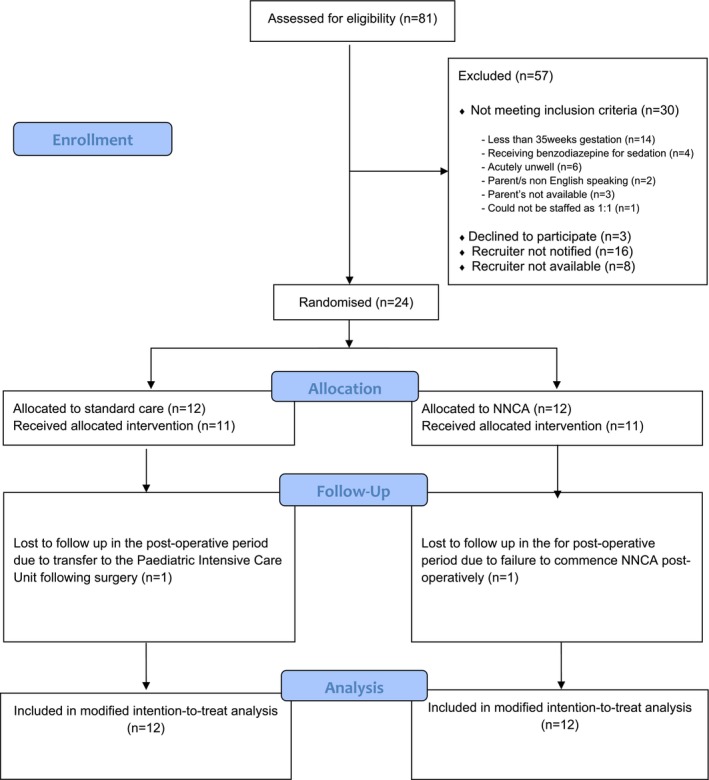
Consort diagram.

During the 10‐month recruitment period, there were 81 surgical infants admitted to the surgical neonatal unit. Fifty‐seven were excluded, 30 of which did not meet eligibility criteria, 16 infants were missed due to the research team not being notified of their admission, three families declined to participate and on eight occasions the researcher was not available to approach families for recruitment, as shown in Figure 2. Due to time restraints and the slow recruitment rate, a decision was made by the research team to cease recruitment prior to obtaining the anticipated 26 participants. A total of 24 families agreed to participate in the trial, with 12 infants randomised into each arm. Following surgery, data from two infants was not included in the post‐operative data analysis due to one infant not receiving the intervention they were randomised to, and the other infant transferring to another health care facility post‐operatively. A third infant receiving the intervention had a medically indicated protocol deviation during the trial as the infant was commenced on a benzodiazepine for additional sedation due to a brief period of instability. Once sedation was ceased (48 h), this infant returned to the designated pathway of the NNCA model as per the NNCA protocol.

### 3.1 Infant Characteristics

Participants in both groups had similar demographics and baseline characteristics (Table 2). Only four (17%) of the 24 infants did not have a surgical condition diagnosed during the antenatal period, and three (13%) infants required transfer from birth hospital to tertiary referral centre for ongoing surgical management. Infants with abdominal wall defects comprised the largest subset of surgical conditions (33%) and more than half of the infants enrolled in the study underwent surgical intervention (67%) within the first 2 days of birth. Other than one infant in the intervention arm not receiving surgery until 20 days of age, time to surgical intervention was shorter for the infants in the intervention arm, with a median time of 29 h compared to 38.5 h in the control arm.

**TABLE 2:** Infant characteristics.

Characteristics	Control (*n* = 12)	Intervention (*n* = 12)
Antenatal diagnosis *n* (%)	8 (40.0)	12 (60.0)
Gestational age at birth, mean (SD) weeks	36.5 (2.0)	37.4 (1.3)
Birth weight, mean (SD) grams	2998 (660.0)	2693 (620.0)
Male *n* (%)	7 (53.8)	6 (46.2)
Inborn *n* (%)	9 (42.9)	12 (57.1)
Resuscitation required *n* (%)	7 (58.3)	5 (41.7)
1 min Apgar score, median (IQR)	9 (6.8–9.0)	8 (7.8–9.0)
Range (min‐max)	2 (7.0–9.0)	4 (5.0–9.0)
5 min Apgar score, median (IQR)	9 (8.0–9.0)	9 (8.8–9.0)
Range (min‐max)	2 (7.0–9.0)	2 (7.0–9.0)
Most frequent diagnosis *n* (%)		
Gastroschisis	3 (25.0)	3 (25.0)
Omphalocele	0 (0.0)	2 (16.7)
Duodenal atresia	2 (16.7)	3 (25.0)
Myelomeningocele	1 (8.3)	2 (16.7)
Other	6 (50.0)	2 (16.7)
Age at time of surgery, median (IQR), hours	38.5 (13.5–49.8)	29.0 (10.8–469)
Gestational age at surgery, mean (SD) weeks	37.7 (1.6)	37.8 (2.0)

### 3.2 Outcomes

#### 3.2.1 Feasibility Outcomes

Overall compliance to all aspects of the trial intervention was achieved for one (9%) infant. Deviations from protocol adherence resulted from incorrect pain scoring frequency in both the immediate 24 h post‐operatively (2 [18%]) and for the 48‐h period following cessation of all analgesics (9 [82%]). There was a failure to reassess infant pain following alterations in analgesia dosage or bolus opioid administration for 10 [91%] infants. Other areas of intervention non‐compliance included: incorrect escalation of analgesia as per designated pathway (3 [27%]), delayed weaning of analgesia (7 [64%]), incorrect prescribing of analgesia (3 [27%]), failure to commence NNCA post‐operatively (1 [9%]) and commencement of benzodiazepine for additional sedation (1 [9%]). Administration of non‐pharmacological measures was documented at least once for all 11 infants in the intervention group. The frequency and type of comfort measure chosen and degree of parental participation was inconsistent and infrequently documented.

The portion of potentially eligible infants not recruited did not meet the feasibility target of less than 20% to progress to a larger RCT. Of the 51 eligible infants admitted during the study period, 24 were missed due to either the researcher not being notified or being unavailable to attend the neonatal unit. The number of infants meeting inclusion criteria was 63%. This result was less than the green light target of 80%, but was within the ‘AMEND’ range of between 50% and 80% meeting criteria that supports modifications for a future larger‐scale trial. All other feasibility criteria outlined a priori met pre‐determined ‘green’ progression criteria (Table 3).

**TABLE 3:** Results of feasibility criteria.

Feasibility criteria outcomes			
	%	%	%
Adherence to NNCA model			9
Number of infants meeting inclusion criteria		63	
Portion of potentially eligible infants not recruited			53
Number of infants recruited (of those approached)	89		
Completeness of data collection/missing data rate	5		
Proportion of participants who were withdrawn from the study	0		
Adverse events	0		

*Note:* 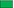, Go; 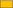, Modify; 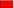, Stop.

Twenty‐two nursing staff members completed the survey, as shown in Table 4. Overall, 19 (95%) staff reported that having formalised guidelines and a clear pathway for escalation of analgesia to meet pain needs was beneficial for managing post‐operative pain. Fourteen (36%) respondents felt that the NNCA model was not easily followed, with a further 16 (27%) indicating the model did not provide greater autonomy to manage pain and discomfort than the standard neonatal pain care in that unit. Three (14%) staff provided additional feedback that the pathways need to be modified and simplified to improve clarity and compliance for any future implementation. Additional information on frequencies and percentages can be found in Appendix S2.

**TABLE 4:** Staff acceptability of NNCA model.

Staff acceptability of NNCA model	Median (IQR)	*n* (%) agree/strongly agree
The NNCA model pathways are logical and able to be followed without difficulty? (*n* = 22)	2 (2–3)	14 (64.0)
Guidelines for use of the NNCA model are comprehensive and contain relevant and appropriate information for holistic management of pain? (*n* = 22)	2 (1–2)	21 (96.0)
It is beneficial to have a formalised escalation pathway for post‐operative pain management? (*n* = 20)	1 (1–1)	19 (95.0)
Management of post‐operative pain is simplified by having a formalised titration and weaning pathway? (*n* = 21)	1 (1–2)	17 (81.0)
The pathways give me greater autonomy in managing infant's comfort and pain? (*n* = 22)	2 (1–3)	16 (73.0)
The pathways improve overall analgesic management in the post‐operative period? (*n* = 22)	2 (1–3)	16 (73.0)

*Note:* Staff acceptability survey where 1 indicates strongly agree to 5 strongly disagree (*n* = 22).

#### 3.2.2 Secondary Outcomes

Secondary outcomes are described in Table 5. Post‐operative pain assessment was completed correctly for two (9%) infants in the study for the period of analgesic therapy and up to 48 h post‐cessation. Eighty‐one per cent (*n* = 9) of infants in the intervention group achieved correct pain scoring assessment for the first 24 post‐operatively compared to 27% (*n* = 3) in the control group. Pain score intensity was less for infants in the intervention group for the first 3 days post‐operatively and up until discharge home or transfer from the surgical unit.

**TABLE 5:** Secondary outcome results.

Secondary outcomes	Control (*n* = 11)	Intervention (*n* = 11)
	Median (IQR)	Median (IQR)
Intensity of pain scores for first 3 days post‐operative	1.9 (0.6–3.1)	1.6 (0.7–2.3)
Intensity of pain scores until transfer or discharge	0.9 (0.3–1.3)	0.6 (0.4–0.8)
Overall opioid consumption, micrograms	1190 (624–4618)	1541 (730–4114)
Time on invasive ventilation, hours	25 (8–40)	39 (17–64)
Time to full enteral feeds, days	12 (7–17)	8 (1–18)
Time to cessation of all analgesics, hours	92 (46–193)	55 (41–91)
Incidence of Iatrogenic withdrawal syndrome	0 (0–0)	0 (0–0)
Length of stay, days	18 (12–29)	13 (9–21)

Duration of opioid therapy was longer for the control group, but total opioid consumption and length of time on ventilatory support was greater for the intervention group. Additionally, both the median time to establishment of full enteral feeds and length of stay in days were shorter for the intervention group infants. Assessment of Iatrogenic withdrawal syndrome using the WAT‐1 scoring tool (Franck et al. 2004) was required in both groups for prolonged administration of opioid therapy (> 5 days). No infant was found to have 100% compliance with the correct frequency of scoring. The four infants meeting assessment for WAT‐1 scoring did not meet scoring thresholds or display any symptoms of Iatrogenic Withdrawal Syndrome.

#### 3.2.3 Parental Satisfaction With Pain Management

Fourteen (64%) families completed the Parent Attitudes About Infant Pain (PAIN) questionnaire prior to their infant's discharge or transfer (Table 6). Parents from both groups reported that they all received verbal information on neonatal pain management. Control group parents reported they received written information more frequently than parents in the treatment group. Independent of group allocation, all families were satisfied with the information they received. Five (71%) parents in the intervention group and six (86%) in the control group reported they were shown at least two different non‐pharmacological comfort measures by nursing staff. This cot‐side education was not reflected in how often families were invited to participate in pain‐relieving strategies across both groups of infants. One parent in the intervention group responded that they were asked to be present to help comfort their infant. No parent in the control group reported that they were asked. There was a notable difference for parents in the intervention group who expected and perceived their infants to experience slightly higher pain intensity than those in the control group. Parents in the intervention group reported more frequently that they preferred not to be present to help comfort their infant during a painful procedure compared to all parents in the control group who would prefer to be present. Overall, parents in both the control and intervention arms of the trial reported being satisfied with infant pain care, effectiveness of analgesia and support given for parental concerns. Additional information on frequencies and percentages for the PAIN questionnaire can be found in Appendix S3.

**TABLE 6:** Parent attitudes about infant pain.

	Control (*n* = 7)	Intervention (*n* = 7)
	Median (IQR)	Median (IQR)
Amount of verbal information about pain control received (1 a lot to 4 none) (*n* = 13)	1.0 (1.0–1.0)	1.0 (1.0–2.0)
Amount of written information about pain control received (1 a lot to 4 none) (*n* = 13)	2.0 (1.0–3.0)	2.0 (2.0–3.0)
Satisfied with information given about pain control (1 very satisfied to 6 very unsatisfied) (*n* = 13)	1.0 (1.0–1.0)	1.0 (1.0–3.0)
Satisfied with care infant received while in the neonatal unit (1 very satisfied to 6 very unsatisfied) (*n* = 14)	2.0 (1.0–2.0)	1.0 (1.0–1.0)
Worst pain infant felt (0 to 10 with nil pain 0 and 10 severe pain) (*n* = 9)	5.5 (4.0–6.0)	7.0 (6.0–8.0)
Expected level of pain that infant would experience (0 to 10 with nil pain 0 and 10 severe pain) (*n* = 13)	7.0 (4.0–8.0)	8.0 (7.0–10.0)
Expected level of pain relief infant would receive (0 to 10 with nil analgesia 0 and 10 substantial amount of analgesia) (*n* = 13)	5.0 (4.0–7.0)	5.0 (3.0–7.0)
Satisfaction that analgesia helped reduce pain (1 very satisfied to 6 very unsatisfied) (*n* = 12)	1.0 (1.0–2.0)	1.0 (1.0–2.0)
Nurses showed parent how to look for signs of pain (from 1, strongly agree, to 6, strongly disagree) (*n* = 13)	2.0 (2.0–3.0)	2.0 (1.0–3.0)
Staff supportive of parent concerns about pain (1, strongly agree to 6, strongly disagree) (*n* = 12)	2.0 (1.0–2.0)	2.0 (1.0–3.0)
Confident staff can tell when infant is in pain (1, strongly agree, to 6, strongly disagree) (*n* = 13)	2.0 (1.0–2.0)	2.0 (1.0–3.0)
Was present during painful procedures (1, never to 4 always) (*n* = 13)	2.0 (1.0–2.0)	2.0 (1.0–2.0)
Asked to be present during painful procedures (1, never to 4 always) (*n* = 13)	2.0 (1.0–2.0)	2.0 (1.0–3.0)

*Note:* Parent Assessment of Infant Nociception (PAIN) survey: Questions reprinted with permission from: Franck et al. (2004).

## 4 Discussion

The aim of this prospective pilot study was to test the feasibility and acceptability of a NNCA pain management model compared to standard clinical care in infants of 35 or more weeks of gestation. Despite not all feasibility criteria reaching the predetermined ‘greenlight’ range for progression to a larger trial, the results provide valuable knowledge on the limitations of the NNCA model and have informed recommendations and changes to the study design for further testing in the future.

Many potential parents of eligible infants were not approached due to both limited availability of research staff outside of office hours and the clinical team failing to notify research staff prior to an eligible infant undergoing surgery. To increase the recruitment rate for a larger scale trial, a modified approach to recruitment is needed. Previous studies have found that having dedicated research staff on site is an important enabler for achieving recruitment targets (Aspden et al. 2021; O'Donnell et al. 2023). Though this increases the opportunity for further families to be approached, it is an additional cost and may not circumvent the problem of recruitment outside of office hours. Alternatively, clinical staff who have received additional training and education on research recruitment and study protocol may be another avenue for increasing recruitment rates. A study by Blewer et al. (2016) found that using bedside nursing staff not only aided in establishing a positive research culture within the unit, but after receiving a 30‐min training session, staff were instrumental in ensuring that desired recruitment rates were achieved in the study timeframe. Though this approach would seem advantageous, particularly as recruitment of surgical infants often needs to be done soon after admission due to the urgency of surgical intervention, clinical priorities and workload restraints may impede the ability for bedside nurses to initiate the consent process (O'Donnell et al. 2023).

Planning for any future trial on the use of this NNCA model should explore the use of advanced consent prior to the infant's admission to the neonatal intensive care unit. Though neonatal surgical conditions are not always known in pregnancy, 83% of infants in this pilot were diagnosed and counselled in the antenatal period. Though some authors have expressed concerns around the additional stress this may place on potential families in the antenatal period (Smyth et al. 2012; Wilman et al. 2015), there are numerous examples in the literature of parents wanting information about neonatal trials during pregnancy in preference to being approached for the first time by the research team in the immediate post‐partum period (McCarthy et al. 2019; Skelton et al. 2024). Additionally, studies examining the validity of the recruitment process in emergent situations have reported that when parents who had consented to neonatal trials soon after delivery were asked 6–12 months post‐discharge information on the trial, only a small number could articulate what the study was about and what intervention their infant received (Ballard et al. 2004). Therefore, not only does obtaining advanced consent for infants known to have a surgical condition antenatally afford parents time to consider their willingness to participate without time pressures in the immediate post‐natal period, it may lessen the burden on research staff being available to consent 24 h per day.

Complete adherence to the trial intervention was not met for 11 (92%) of the 12 infants randomised to the intervention arm. Despite the failure of one infant starting on the NNCA model in the post‐operative period, the remaining 10 (83%) infants did complete elements of the model accurately, most notably pain scoring in the first 24‐h period. Comparatively, only three (27%) infants in the control arm received correct pain score assessment for the first post‐operative day. Though there was limited documentation for medically indicated pathway deviations, noted variances included decisions by the bedside nurse to either delay weaning or request an early review by the medical team for increased analgesia outside the suggested parameters. This pattern of delayed weaning and non‐adherence to the model was consistently unsupported by reported pain scores and appeared to be in response to non‐pain‐related factors such as the need to maintain a secure airway in ventilated infants, hunger and other care requirements of an infant recovering from surgery.

Compliance with protocols and guidelines is challenging. Workload restraints, patient acuity and protocol complexity have all been identified as barriers to adherence (Sinuff 2007). There is a paucity of research on the use of pain protocols and non‐compliance in the literature. Studies on protocols and guidelines for sedation in both neonates and children suggest that though they are beneficial for improving clinical outcomes, overall adherence is poor (Aukes et al. 2015; Keogh et al. 2015). A review by Burns in 2012 found total compliance rates for protocols managing sedation in several adult intensive care units ranged from 8% to 28%. Further to this, the review suggests that tools that require frequent revision and complex decision‐making steps are less likely to be followed as they may be seen by the bedside clinician as a competing priority that increases workload and impedes independent decision making (Burns 2012). This is congruent with the feedback received on the acceptability and usability of the NNCA model, where only 64% of bedside clinicians reported the model easy to follow. Additional free text comments also suggest the pathways to be ‘wordy’ and ‘busy looking’ requiring multiple read‐throughs to ensure correct interpretation.

Though the aim of this NNCA model was to improve pain relief by individualising analgesia requirements and allowing nursing staff to titrate dosage based on documented pain scores, 27% of respondents to the staff survey did not believe that the NNCA model provided greater autonomy to manage post‐operative pain. A study by Flynn and Sinclair (2005) examining protocol use in an Irish intensive care unit, found that although the nursing staff could agree upon the benefits of a protocol to help guide practice, it could not replace professional judgement and experience. Similar sentiments were described by Macpherson et al. (2024) where bedside clinicians felt protocols impinged upon their autonomy and restricted their scope of practice as critical care nurses. Managing sedation and analgesia is often more complex than following a guideline, and patient and environmental factors need to be considered when making decisions. A NNCA model that requires neonatal nurses to titrate analgesia based on documented pain scores and pre‐determined timeframes may account for the overall poor compliance and reluctance to wean analgesia in this study. Nurses participating in this pilot were accustomed to weaning opioid therapy within a pre‐prescribed range and may have found the model impinged upon their ability to use their clinical judgement to decide on the analgesic needs of the infant. This ability of nurses to wean analgesia independently is not standard care across Australasian surgical neonatal units (Muirhead et al. 2023) therefore, neonatal nurses in other units caring for post‐operative infants may find the opportunity to titrate analgesia without a need for repeated medical review advantageous, with greater adherence to the intervention protocol.

The data obtained on the secondary outcomes, including parental perception of neonatal pain management, were feasible as potential primary outcomes for a future larger scale trial. Though assessing effectiveness was not the aim of the study, a trend towards reaching full enteral feeds earlier, shorter duration of analgesics, reduced length of stay and lower pain scores was evident in infants receiving the intervention arm. Despite the positive trend observed with these findings, the secondary outcome findings should be interpreted with caution due to the small sample size used in this pilot study. A higher cumulative dose of opioids and longer duration on invasive respiratory support is incongruent with what would typically be expected in infants ceasing opioid therapy earlier than their counterparts (Bhandari et al. 2005; Keane et al. 2024). No infant in either group developed IWS or had any adverse events attributed to analgesic administration or pain management during the trial. Though the NNCA model has a strong focus on parental involvement and encourages families to be present and deliver non‐pharmacological measures as first line analgesic therapy, there was limited evidence that this occurred, particularly in the early post‐operative period. Previous studies have suggested that parents would prefer to be involved in providing comfort to their infant (Franck et al. 2012) and should be actively involved in pain assessment and management (Campbell‐Yeo et al. 2024). This is congruent with the results of the parent survey in which 77% of respondents stated that they would prefer to be present during painful procedures to comfort their infant. Despite this, parents in the intervention group reported that they were rarely asked to be present to offer comfort measures.

Parental presence in the neonatal unit in the early post‐partum period is influenced by several factors: method of delivery, birthing location, maternal well‐being or childcare commitments (Ranu et al. 2021). As 67% of infants in the study underwent operative intervention within the first 48 h post‐partum, parents may have had limited ability to be present to provide comfort during this early post‐operative period as a result of their own recovery, birthing at another health care facility or need to care for other children in the family. Furthermore, bedside clinicians may have been reluctant to ask families to be present at the cot‐side during this time due to either an under appreciation of the benefits of parental delivered comfort measures or a desire to support parents' recovery and rest. Though parents need appropriate time and resources to recover from the birth and adjust to the neonatal intensive environment, it is also paramount that both staff and parents are aware of the significant benefits of parental delivered comfort interventions (Ullsten et al. 2021), both in the immediate post‐operative period and throughout the infant's intensive care recovery.

### 4.1 Study Limitations

This pilot was conducted in a single neonatal surgical centre. As there is no current gold standard for neonatal post‐operative pain management, variability between different neonatal units may have resulted in differences in outcome data with the implementation of a model of NNCA. Secondly, as this was an unblinded study, inherent biases on the part of both nurses and families may have been present in the delivery of the intervention. Furthermore, though this study was underpowered for effectiveness, statistical analysis was performed and differences between groups and trends noted to help guide the development of a larger RCT in the future.

## 5 Conclusion

The findings of this pilot trial suggest that a larger RCT assessing the effectiveness of NNCA in the surgical neonate would require modifications to the model and to the trial protocol to improve feasibility. Having an opportunity to discuss the trial and obtain parental consent in the antenatal period or extending the inclusion criteria to infants less than 35 weeks gestation may provide further opportunities to increase future recruitment rates. To improve protocol adherence, including increased parental participation, targeted staff education, consideration of a different trial site and simplification of the model that facilitates increased flexibility for acute care nurses to integrate and adapt pain relieving strategies based on not only pain scores but the overall status of the post‐operative infant would be warranted for any future research on the use of a model of NNCA.

## Conflicts of Interest

The authors declare no conflicts of interest.

## Supporting information


Appendix S1.

Appendix S2.

Appendix S3.

Appendix S4.

Appendix S5.

## Data Availability

The data that support the findings of this study are available from the corresponding author upon reasonable request.
