# Supplementary material for: A Neonatal Nurse‐Controlled Model of Analgesia to Manage Post‐Operative Pain in the Surgical Neonate: A Pilot Randomised Controlled Trial
Source: J Adv Nurs. 2025 Apr 24;82(2):1725–36. doi: 10.1111/jan.16992 (PMC12810603; doi:10.1111/jan.16992)
Supplement: Supplementary file 1 — Appendix S1. [file JAN-82-1725-s001.docx]

**APPENDIX III: Parent attitudes about infant pain (frequencies and percentages)**

|  | **Scale** | **Control**  **n (%)** | **Intervention**  **n (%)** |
| --- | --- | --- | --- |
| *Amount of verbal information about pain control received (1 a lot to 4 none), (n=13)* | 1  2  3  4 | 6(85.7)  1(14.3)  0  0 | 4(66.7)  1(16.7)  0  0 |
| *Amount of written information about pain control received (1 a lot to 4 none), (n=13)* | 1  2  3  4 | 2(28.6)  3(42.9)  1(14.3)  1(14.3) | 0  4(66.7)  1(16.7)  1(16.7) |
| *Satisfied with information given about pain control (1 very satisfied to 6 very unsatisfied), (n=13)* | 1  2  3  4  5  6 | 6(85.7)  1(14.3)  0  0  0  0 | 4(66.7)  0  2(33.3)  0  0  0 |
| *Satisfied with care infant received while in the neonatal unit (1 very satisfied to 6 very unsatisfied), (n=14)* | 1  2  3  4  5  6 | 3(42.9)  4(57.1)  0  0  0  0 | 6(85.7)  1(14.3)  0  0  0  0 |
| *Worst pain infant felt (0 to 10 with nil pain 0 and 10 severe pain), (n=9)* | 0  1  2  3  4  5  6  7  8  9  10 | 0  0  0  1(25.0)  0  1(25.0)  2(50.0)  0  0  0  0 | 0  0  0  0  0  0  2(40.0)  2(40.0)  1(20.0)  0  0 |
| *Expected level of pain that infant would experience (0 to 10 with nil pain 0 and 10 severe pain), (n=13)* | 0  1  2  3  4  5  6  7  8  9  10 | 0  0  0  0  3(42.9)  1(14.3)  1(14.3)  2(28.6)  0  0  0 | 0  1(16.7)  0  0  1(16.7)  2(33.4)  1(16.7)  0  0  0  1(16.7) |
| *Expected level of pain relief infant would receive (0 to 10 with nil analgesia 0 and 10 substantial amount of analgesia, (n=13)* | 0  1  2  3  4  5  6  7  8  9  10 | 0  0  0  0  3(42.9)  1(14.3)  1(14.3)  2(28.6)  0  0  0 | 0  1(16.7)  0  0  1(16.7)  2(33.4)  1(16.7)  0  0  0  1(16.7) |
| *Satisfaction that analgesia helped reduce pain (1 very satisfied to 6 very unsatisfied), (n=12)* | 1  2  3  4  5  6 | 4(66.7)  2(33.3)  0  0  0  0 | 5(83.3)  0  1(16.7)  0  0  0 |
| *Nurses showed parent how to look for signs of pain (from 1, strongly agree, to 6, strongly disagree), frequency (f), percentage(%), (n=13)* | 1  2  3  4  5  6 | 0  5(71.4)  2(28.6)  0  0  0 | 2(33.3)  2(33.3)  1(16.7)  0  0  0 |
| *Staff supportive of parent concerns about pain (1, strongly agree to 6, strongly disagree), frequency (f), percentage(%), (n=12)* | 1  2  3  4  5  6 | 3(50.0)  3(50.0)  0  0  0  0 | 3(50.0)  1(16.7)  2(33.3)  0  0  0 |
| *Confident staff can tell when infant is in pain (1, strongly agree, to 6, strongly disagree), frequency (f), percentage(%), (n=13)* | 1  2  3  4  5  6 | 3(42.9)  4(57.1)  0  0  0  0 | 2(33.3)  3(50.0)  1(16.7)  0  0  0 |
| *Was present during painful procedures (1, never to 4 always), frequency (f), percentage(%), (n=13)* | 1  2  3  4 | 2(28.6)  5(71.4)  0  0 | 3(50.0)  2(33.3)  1(16.7)  0 |
| *Asked to be present during painful procedures (1, never to 4 always), frequency (f), percentage(%), (n=13)* | 1  2  3  4 | 2(28.6)  5(71.4)  0  0 | 3(50.0)  1(16.7)  2(33.3)  0 |

Parent Assessment of Infant Nociception (PAIN) survey: Questions reprinted with permission from: Franck LS, Cox S, Allen A, Winter I. Parental concern and distress about infant pain. Arch Dis Child Fetal Neonatal Ed. 2004 Jan;89(1):F71-5
