# Supplementary material for: A Neonatal Nurse‐Controlled Model of Analgesia to Manage Post‐Operative Pain in the Surgical Neonate: A Pilot Randomised Controlled Trial
Source: J Adv Nurs. 2025 Apr 24;82(2):1725–36. doi: 10.1111/jan.16992 (PMC12810603; doi:10.1111/jan.16992)
Supplement: Supplementary file 2 — Appendix S2. [file JAN-82-1725-s002.docx]

Supplementary Material Appendix I

Neonatal Nurse Controlled Analgesia for the Post-operative Infant

- The goal of Neonatal Nurse Controlled Analgesia (NNCA) in the post-operative infant is to provide a responsive mode of analgesia that alleviates pain and discomfort while minimizing the unwanted effects of excessive analgesic use.
- The following sections/headings of the NNCA guideline outline the overarching principals of the model, providing key information required for clinical application in the neonatal intensive care unit.
- The final section of the guideline provides three (3) algorithms to be used by the direct care nursing clinician to assess and manage pain in the post-operative period.

## Patient selection

Eligibility criteria:

- - Infants ≥35weeks (PMA)
  - Opioid naïve infants or infants commenced on opioids for the first time within the previous 48hrs
  - Hemodynamically stable
  - Cared for in a 1:1 nurse allocation ( for at least the first 24hrs post-operatively) by a nursing clinician with at least 2 years neonatal surgical experience
  - Neonatologist approved Exclude Infants:
  - < 35weeks gestation (PMA)
  - Any Illness complicated by physical instability (e.g. Persistent Pulmonary Hypertension of the Newborn ) in which additional sedation is required to manage clinical condition.
  - Complex surgical conditions (as determined by treating neonatologist)
  - Impaired hepatic or kidney function/Unconjugated hyperbilirubinemia

## Families

Family members are integral to providing optimal pain management. Encourage parental presence as able and provide families with appropriate parent education resources. Parents are often best placed to know their baby’s cues and provide valuable information about their infant’s pain

experience. Infant’s may also be comforted and positively respond to parental voice and scent. The use of other non-pharmacological measures may also be implemented by parents/family members under the guidance of the primary care nurse.

## Comprehensive Pain Assessment

Pain and discomfort will be assessed on a continual basis. Scoring using a validated pain assessment tool (e.g. MPAT/NPASS/PIPP) will occur hourly at a minimum in the first 24 hours post-operatively. After this time, assessment will be as per designated pathway of NNCA or more frequently if indicated by clinical condition. Pain scores will guide the administration and titration of pharmacological agents. Scoring may be performed more frequently if required. Re-assessment should also occur following any pharmacological/non-pharmacological intervention to measure effectiveness and need for further interventions. Parental input regarding their infant’s cues and responses should also be considered alongside pain assessment scores when determining analgesic needs. Irritation caused by hunger/intubation/noxious external stimuli may also contribute to elevated pain scores.

Consideration of all factors that may cause discomfort must be evaluated prior to escalation of pharmacological analgesia.

## Non-pharmacological measures (NPM’s)

Optimal treatment of pain and agitation in neonates requires a multimodal approach that includes the use of non-pharmacologic strategies. The use of non-pharmacological measures (NPM) should be considered as important adjunctive therapy for the surgical infant. Exploration of underlying sources of discomfort and other contributing environmental factors should be considered with modification as appropriate prior to any escalation in pharmacological support.

Measures to be utilized for the post-operative infant are;

- - Decrease environmental stimulus by shielding eyes from direct light, not talking over infant, use of eye shades or ear muffs as appropriate
  - Containment using boundaries, a wrap, and/or hand hugs
  - Swaddling as appropriate (dependent on clinical condition and surgical repair site)
  - Facilitated tucking and positioning
  - Maternal/paternal voice
  - Non-nutritive sucking with pacifier (with parental consent)
  - Scent hearts (maternal/paternal scent) if appropriate
  - Positive touch
  - Nappy changes
  - Skin to therapy (as appropriate with consideration of type of surgery and time elapsed since surgery).

## Pharmacological agents

Morphine is the preferred choice of opioid therapy in the surgical infant, and will be the only opioid utilized in the NNCA model. Opioids are associated with respiratory depression, hypotension, reduced gastro-intestinal motility and dependence ^2^ and must be administered judiciously.

Intravenous paracetamol is to be initiated within the first two (2) hours post-operatively (if not administered in previous 6hrs) and will continue based on individual analgesic needs of the infant. Babies with a history of Impaired hepatic or kidney function/Unconjugated hyperbilirubinemia will be excluded from using this NCCA model. Sucrose 24% will be utilized for procedural pain when required.

A loading dose of morphine may be administered prior to the commencement of an opioid infusion depending on choice, dose and time administered of pharmacological agent/s at completion of surgical procedure. Need for loading dose will be determined by treating team prior to commencement of NNCA. Suggested dosing for NNCA is 50-100μg/kg.

Dexmedetomidine may be initiated as adjunctive pharmacological therapy in some surgical neonatal units in the post-operative period following medical/NNP review. If benzodiazepines (i.e. midazolam) are required to aid in further sedation of the infant, or dexmedetomidine is required outside of this defined dosing range, the NNCA model will be ceased and analgesic management will continue as per individual unit protocol. A complete list of pharmacological agents/dosages/parameters used in the NNCA model are as below:

| **Pharmacological agent** | **Route** | **Minimum dosing** | **Maximum dosing** | **Loading dose** | **Comments** |
| --- | --- | --- | --- | --- | --- |
| **Morphine** | IV  continuous Infusion | 1μg/kg/hr | 30μg/kg/hr (15μg/kg/hr for non-intubated  infants) | 50-  100μg/kg | Usual rate 5- 20μg/kg/hr |
| **Morphine** | IV Bolus | 25μg/kg | 75μg/kg | - | 75μg/kg maximum dose in a 4hr period |
| **Dexmedetomidine *** | IV infusion | 0.1μg/kg/hr | 0.3μg/kg/hr | - | No bolus dosing |
| **Paracetamol** | IV | 7.5mg/kg 6^th^  hrly | 7.5mg/kg 6^th^ hrly | - | 40mg/kg per day  maximum dose |
| **Sucrose 24%** | Oral | 0.1mls | 1ml per event | - | Maximum of 5mls per day for  procedural pain |

**Table 1: Neonatal Nurse Controlled Analgesia pharmacological parameters**

* Pharmacological adjunct available if supported by local context.

## Non- intubated infants

If infant is not receiving invasive respiratory support, use opioid therapy with caution. NNCA upper limit for opioid therapy for non-intubated infants is 15μg/kg/hr. If non-intubated infant continues to score in the ***SEV*** range despite receiving maximal morphine infusion of 15μg/kg/hr, immediate medical/NNP review is required. Consider need for escalation in respiratory support to increase pharmacological administration. If infant remains non-intubated, any incremental increase in morphine rate or bolus dosing will only occur after ongoing medical/NNP review. If other pharmacological agents are able to be utilized as per local context, dexmedetomidine may be considered as its use is not associated with respiratory depression.

## Monitoring

All infants receiving NNCA for post-operative pain management, will be cared for in a neonatal intensive care unit. Access to emergency resuscitation equipment and the availability of skilled staff with the ability to provide advanced respiratory support if required 24 hours per day. The infant will require continuous cardio-respiratory and oxygen saturation monitoring, including 2 to 4hrly invasive/non-invasive blood pressure monitoring.

## Opioid Rotation/switch

Opioid rotation will only occur if there is an escalation beyond the NNCA dosage limits to reach effective pain relief or an adverse reaction observed to current opioid. A neonatologist review must precede an opioid rotation. The NNCA will be discontinued if an opioid switch occurs. If an infant requires a change from IV morphine to oral morphine administration, the ratio for conversion of IV morphine to oral morphine will be 1:2^1^.

## NCA delivery

- The NNCA will be prescribed using defined parameters as per table 1.
- Commence on NNCA Path A. Transition to Path B after 12 hours if pain scores are in the ***LOW-MOD*** range, otherwise continue on path A until ***LOW*** pain scores are achieved for 4- 6hrs. After this, transition to Path B. If infant still requiring opioid therapy at day five (5) post-operative, transition to Path C.
- A MO/NNP review can be requested at any stage of the NNCA but mandatory reviews are required and specified on each pathway to assess current clinical condition of infant prior to further escalation in management.
- All rate changes and administration of bolus medications as per NNCA, will be independently double checked by two (2) registered nurses/midwives/medical officers.
- Hourly assessment and documentation of IV administration site for patency.
- Hourly documentation of dose infused for previous hour.
- Any non-pharmacological/pharmacological interventions must be clearly documented on infant’s standard observation/fluid administration/pain assessment tool or clinical form as per local context.
- If NNCA discontinued due to escalation in pharmacological management beyond parameters or deterioration in infant’s clinical condition, NNCA may be re-commenced once pharmacological agents have returned to NNCA parameters and/or infant’s clinical condition permits following discussion with treating team.

## Weaning

If the infant remains on opioid therapy > 5 days, assessment of IWS must be initiated using relevant withdrawal tool. Though Iatrogenic withdrawal scores aid in the detection of Iatrogenic Withdrawal Syndrome (IWS), individual iatrogenic withdrawal scores are not used in isolation to dictate management. Consecutive withdrawal scores, clinical condition, pain scores, and other factors such as hunger/intubation/noxious external stimulus should all be considered in the diagnosis of Iatrogenic withdrawal syndrome and subsequent weaning of analgesic support. Please refer to the instructions/protocol for designated withdrawal tool used in each individual unit to aid in the detection of IWS. A diagnosis of IWS will occur following review by the treating multi-disciplinary team. If adjunctive pharmacological therapy used (i.e. Dexmedetomidine), weaning should be done concurrently with opioid weaning.

## Abbreviations

NNCA: Neonatal Nurse Controlled Analgesia NPM: Non -Pharmacological Measure MPAT: Modified Pain Assessment Tool NPASS: Neonatal Pain and Sedation Scale PIPP: Premature Infant Pain Profile

WAT-1: Withdrawal Assessment Tool NAS: Neonatal Abstinence Score

IWS: Iatrogenic Withdrawal Syndrome MO: Medical Officer

NNP: Neonatal Nurse Practitioner N/A: Non -Applicable

## References

1. Queensland Clinical Guidelines (August 2019) *Neonatal Medicines.* [internet]. Cited 3^rd^ June 2022. Available from: htts://w.health.qld.gov.au/ data/assets/pdf_file/0043/859678/g- neomed.pdf
2. Bellù, R., Romantsik, O., Nava, C., Waal, K. A., Zanini, R., & Bruschettini, M. (2021). Opioids for newborn infants receiving

# Neonatal Nurse Controlled Analgesia (NNCA for the Post-Operative Infant: Path A)

Commence NNCA in yellow box of Path A. If pain scores remain in the ***LOW -MOD*** range after 12hrs, transition to Path B for continued management. If scores are in the ***SEV*** range, continue on Path A until ***LOW*** scores achieved or NNCA suspended as per designated pathway. Document and date all variances.

***LOW*** score: MPAT ≤ 4,

***MOD*** score:

MPAT 5-9,

***SEV*** score: MPAT ≥10,

Cycle

1. 

2. 

**Review** 

3. 

4. 

**Review** 

5. 

6. 

**Review** 

7. 

8. 

**Review** 


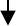


A

Achieving scores

**MPAT <10**

- Consider need for loading dose of morphine 50-100μg/kg (following MO/NNP review)
- Commence continuous morphine infusion at 10μg/kg/hr
- Employ NPM’s as per NNCA
- Administer bolus 25μg/kg of morphine q15min during first hour to a max of 75μg/kg to achieve MPAT ≤ 4. If 3 x bolus needed, increase infusion by 5mcg/kg/hr.
- IV Paracetamol 7.5mg/kg to be commenced within 2hrs of commencing on Pathway A if not

administered in previous 6hrs

- Reassess pain scores in 60mins

Date and time commenced

Pathway A

____________

Yes No

***LOW* pathway**

**MPAT ≤ 4**

- Continue current analgesia.
- Encourage parental presence & NPM measures as per NNCA.
- Administer paracetamol if not given in previous 6 hrs.
- Return to “A” and reassess scores every 60mins.

**Adjunctive pharmacological**

Commence dexmedetomidine 0.2-0.3μg/kg/hr. If further increases in adjunctive pharmacological therapy required, cease NNCA and manage pain as per standard unit protocol.

***MOD* pathway**

**MPAT 5-9**

- Encourage parental presence.
- Explore other potential sources of pain.
- Implement NPMs as per NNCA.
- Administer paracetamol if not given in previous 6 hrs.
- Return to “A” and reassess scores

every 30-60 mins.

**End of cycle**

- MO/NNP review is **REQUIRED** if remains in ***MOD*** range after **completing** two cycles through this pathway.
- Increase morphine infusion by 5μg/kg/hr after MO/NNP review
  - If baby not intubated

***morphine*** infusion must not exceed 15μg/kg/hr without medical/NNP review

- - If not intubated and

requiring 15μg/kg/hr discuss with MO/NNP regarding intubation.

- - If baby is intubated then a maximum of 20μg/kg/hr must not be exceeded in ***MOD*** pathway.
- If more 20μg/kg/hr of morphine is required then escalate to the ***SEV*** pathway.

***SEV* pathway**

**MPAT ≥ 10**

- Encourage parental presence.
- Implement NPMs as per NNCA.
- Explore other potential sources of pain.
- Administer paracetamol if not given in previous 6 hrs.
- Give bolus 25μg/kg of morphine q15 min up to a max of 75μg/kg (in 4hrs) as required
- Consider MO/NNP review.
- Return to “A” and reassess pain score every 30-60mins.

**End of cycle**

- - If remains in ***SEV*** range after **completing two** cycles through this pathway, increase morphine infusion by 5μg/kg/hr.
  - Return to “A” and reassess pain score in

30-60mins.

- - If remains in ***SEV*** range after **completing three** cycles through this pathway, MO/NNP **required.**
  - Consider increasing morphine infusion by 5μg/kg/hr. Return to “A” and reassess pain score every 30-60mins.
  - If baby not intubated, morphine infusion must not exceed 15μg/kg/hr. If not intubated and requiring greater than 15μg/kg/hr, discuss with MO/NNP regarding intubation.
  - If baby is intubated then a maximum of 30μg/kg /kg/hr must not be exceeded.
  - If remains in ***SEV*** range after **completing**

**four** cycles through this pathway, increase morphine infusion by 5μg/kg/hr.

- - MO/NNP review **REQUIRED.**
  - Return to “A” and reassess pain score in

30-60mins.

- If baby requiring 30μg/kg/hr of morphine and remains in ***SEV*** range, then consider adjunctive pharmacological therapy as per NNCA following MO/NNP review
- If > 30μg/kg/hr of morphine is required, suspend NNCA and manage as per standard

unit protocol.

Cycle

1. 

2. 

3. 

**Review **

4. 

**Review **

5. 

**Review **

6. 

**Review** 

Discontinue. 

**If after 12hrs, infant achieving *MOD* scores or lower, transition to Path B**

# Neonatal Nurse Controlled Analgesia (NNCA) for the Post-Operative Infant:

**Path B (weaning < 5 days)**

1. Use Path B from 12hrs -4 days post-operatively if achieving ***MOD*** pain scores or lower.
2. Transition to Path C if infant requiring analgesia > 5days


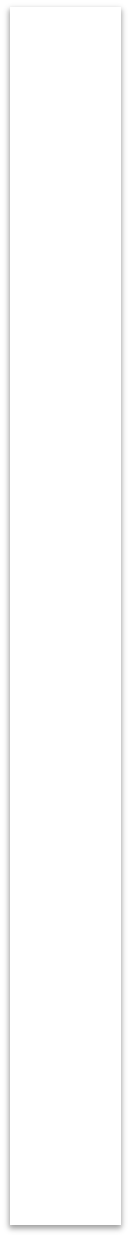

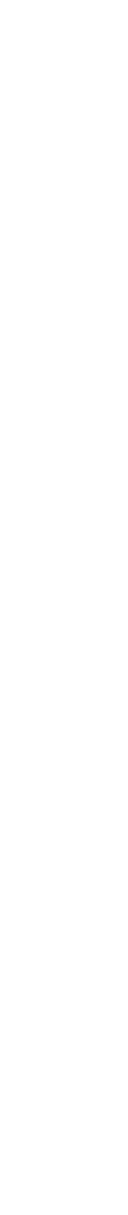


At ≥ 12hrs post-operative

achieving scores

B

**MPAT ≤ 4**

Date and time commenced

Pathway B

__________________ _______

**Pain score assessment**

***LOW*** score: MPAT ≤ 4,

***MOD*** score: MPAT 5-9,

***SEV*** score: MPAT ≥10,

Yes No

Cycle

1. 

2. 

3. 

**Review** 

4. 

**Review** 

1. 

2. 

3. 

**Review** 

4. 

**Review** 

***SEV* pathway**

**MPAT ≥ 10**

- Pause/do not commence weaning.
- Encourage parental presence.
- Assess for other contributing factors.
- Implement NPM’s as per NNCA.
- Administer paracetamol if not given in previous 6 hrs.
- Return to “B” and reassess in

30 -60mins.

**End of cycle**

- If after **completing** two cycles through ***SEV*** pathway, request MO/NNP review and return to Path A for continued assessment and management.

Cycle

1. 

2. 

**Review** 

1. 

2. 

**Review** 

***MOD* pathway**

***LOW* pathway**

**MPAT ≤ 4**

- Wean opioid rate by 20% of highest dose every 4-6hours until ceased.
- Once opioid infusion ceased, reassess need for continuation of regular paracetamol.
- Return to “B “ and reassess every 60mins for at least 24hrs post-operatively.

**End of cycle**

- If scores remain in this range after this time, pain score every 2-4hrs until all analgesics ceased for 48hrs.

**MPAT 5-9**

- - Pause/do not commence weaning.
  - Encourage parental presence.
  - Assess for other contributing factors.
  - Implement NPM’s as per NNCA.
  - Administer paracetamol if not given in previous 6 hrs.
  - Return to “B” and reassess in 30 - 60mins

**End of cycle**

- If after **completing** two cycles

through ***MOD*** pathway,

consider returning analgesia to previous dose if weaning

commenced.

- Return to “B” and reassess pain score in 30-60mins.

- If pain scores remain in ***MOD*** range after **completing** three cycles through pathway, return analgesia to previous dose if not done previously.

**Weaning adjunctive pharmacological therapy**

- If <24hrs, cease dexmedetomidine without weaning.
- If >24hrs, reduce rate by 50%. If well tolerated, cease infusion 4- 6hrs. Return to “B “& reassess in 60mins.
- Return to “B” and reassess.

pain score in 30-60mins.

- If analgesia has already been increased after 2^nd^ cycle, request MO/NNP review & consider use of adjunctive

pharmacological therapy as

per NNCA.

- Return to “B” and reassess pain score in 30-60mins.
- If after **completing** four cycles infant remains in ***MOD*** range, return to Path A and request MO/NNP review.

# Neonatal Nurse Controlled Analgesia (NNCA) for the Post-Operative Infant: Path C (weaning >5 days)

a. Use Path C if the infant is requiring opioid analgesia 5 days post-operatively

**Pink path**

**MPAT ≤ 4**

- Do not wean.
- Maintain current opioid rate.
- Encourage parental presence.
- Implement NPM’s as per

NNCA.

- Consider MO/NNP review.
- Continue to score pain 2- 4hrly and IWS as per tool protocol. May reassess IWS more frequently if required.
- Return to “C” to reassess

eligibility to wean in 4-6hrs.

**End of cycle**

- After **completing** two cycles through pink pathway, a MO/NNP review is **REQUIRED**
  - Consider increasing opioid rate to previous dose/administer hourly rate as a bolus.
  - If opioid rate increased, maintain rate for 24hrs before considering further wean.
  - Continue to score IWS as per tool protocol or more frequently if required.
- After **completing** three cycles through pink pathway, a MO/NNP review is **REQUIRED**
  - If IWS scores remain in this range despite increase in rate or administration of bolus dose, discontinue NNCA and manage IWS as per treating medical team.

Cycle

1. 

2. 

**Review** 

3. 

**Review** 

1. 

2. 

**Review** 

3. 

Discontinue. 

**Blue path**

**MPAT ≥ 5**

- Do not wean.
- Maintain current opioid rate.
- Encourage parental presence.
- Implement NPM’s as per

NNCA.

- Assess for other contributing factors.
- Consider MO/NNP review.
- Return to “C” and reassess pain score in 60mins and IWS as per tool protocol.

**End of cycle**

- After **completing** two cycles through blue pathway, a MO/NNP review is **REQUIRED**

Increase opioid rate to previous dose/administer hourly rate as a bolus.

- - If opioid rate increased, maintain rate for 24hrs before considering further wean.
  - Continue to score IWS as per tool protocol or more frequently if required.
- After **completing** three cycles through blue pathway, a MO/NNP review is **REQUIRED**
  - Consider opioid rate increase/administer hourly rate as bolus
  - Reassess MPAT and WAT-1scores in 60mins
  - If IWS & pain scores remain in this range, discontinue NNCA and manage as per treating medical team.

Cycle

1. 

2. 

**Review** 

3. 

**Review** 

1. 

2. 

**Review** 

3.

Discontinue.



At > 5 days post-operative achieving withdrawal scores

**WAT 1 <3**

**NAS <8**

C

At ≥ 5 days post-operative achieving WAT-1

Score ≤ 3

C

c

No

Yes

**Green path**

**MPAT ≤ 4**

- Wean opioid rate by 20% of highest dose once per day until opioid ceased or changed to enteral therapy as per Neonatologist.
- Continue to pain score 2-4hrly and IWS score as per IWS tool protocol.

- May score IWS more frequently if required.

**Pain & IWS scores**

***LOW*** score: MPAT ≤ 4,

***MOD*** score: MPAT 5-9,

***SEV*** score: MPAT ≥10,

**WAT 1**: Score >3 IWS

**Purple path**

**MPAT ≥ 5**

- Do not wean
- Maintain current opioid rate.
- Assess for other contributing factors.
- Encourage parental presence.
- Implement NPM’s as per

NNCA.

- Continue to score IWS as per tool protocol.
- Consider MO/NNP review.
- Return to “C” and reassess pain score in 60mins

**End of cycle**

- After **completing** two cycles through purple pathway, a MO/NNP review is **REQUIRED**
  - If pain scores remain in ***MOD*** range, consider weaning opioid rate by 10% of the highest dose once per day.
  - Continue to pain score 2-4hrly or more frequently if required.
  - If scores remain in ***SEV*** range, continue to pause weaning, return to “c” and reassess in 60mins.
- If After **completing** three cycles through purple pathway, a MO/NNP review is **REQUIRED**
  - If scores remain in ***SEV*** range, discontinue NNCA and manage as per treating medical team.

Cycle

1. 

2. 

**Review** 

3. 

**Review** 

1. 

2. 

**Review** 

3. 

Discontinue. 

Date and time commenced

Pathway C

__________________ _______
