# Supplementary material for: A Neonatal Nurse‐Controlled Model of Analgesia to Manage Post‐Operative Pain in the Surgical Neonate: A Pilot Randomised Controlled Trial
Source: J Adv Nurs. 2025 Apr 24;82(2):1725–36. doi: 10.1111/jan.16992 (PMC12810603; doi:10.1111/jan.16992)
Supplement: Supplementary file 3 — Appendix S3. [file JAN-82-1725-s005.docx]

**
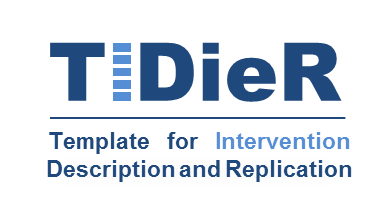
The TIDieR (Template for Intervention Description and Replication) Checklist*:**

Information to include when describing an intervention and the location of the information

| **Item number** | **Item** | **Where located **** | |
| --- | --- | --- | --- |
|  |  | Primary paper  (page or appendix  number) | Other ^†^ (details) |
|  | **BRIEF NAME** |  |  |
| **1.** | Provide the name or a phrase that describes the intervention. | Page 6. | Line 154 |
|  | **WHY** |  |  |
| **2.** | Describe any rationale, theory, or goal of the elements essential to the intervention. | Page 3-4 | Introduction |
|  | **WHAT** |  |  |
| **3.** | Materials: Describe any physical or informational materials used in the intervention, including those provided to participants or used in intervention delivery or in training of intervention providers. Provide information on where the materials can be accessed (e.g. online appendix, URL). | Model of NNCA (intervention) found in supp material Appendix 1 |  |
| **4.** | Procedures: Describe each of the procedures, activities, and/or processes used in the intervention, including any enabling or support activities. | NNCA model in supp material Appendix 1 | Also lines 154-189 |
|  | **WHO PROVIDED** |  |  |
| **5.** | For each category of intervention provider (e.g. psychologist, nursing assistant), describe their expertise, background and any specific training given. | Neonatal nurse with a minimum of 2yrs neonatal surgical experience | Page 4, line 118 |
|  | **HOW** |  |  |
| **6.** | Describe the modes of delivery (e.g. face-to-face or by some other mechanism, such as internet or telephone) of the intervention and whether it was provided individually or in a group. | Intervention delivered by the nurse caring for the baby post-operatively. | Lines 117-118 and lines 154-189 and Appendix 1 NNCA model |
|  | **WHERE** |  |  |
| **7.** | Describe the type(s) of location(s) where the intervention occurred, including any necessary infrastructure or relevant features. | Cardiac/surgical neonatal intensive care unit in Brisbane, Australia. | Page 4: Lines 107-109 |
|  | **WHEN and HOW MUCH** |  |  |
| **8.** | Describe the number of times the intervention was delivered and over what period of time including the number of sessions, their schedule, and their duration, intensity or dose. | The NNCA model was utilised to guide post-operative pain management from the time the infant returned from theatre until 48hrs post cessation of opioid therapy. | An amendment has been made to page 6: line 184 to make this clearer. |
|  | **TAILORING** |  |  |
| **9.** | If the intervention was planned to be personalised, titrated or adapted, then describe what, why, when, and how. | The entire model is based on the infant’s pain scores and time elapsed post operative. The three different pathways clearly outline when to transition to the next depending on time when the infant retuned to the ward and the infants score. A summary is provided under the Intervention heading under 2.4 Protocol. All interventions are based on assessed score as per the NNCA model in supp material Appendix I | Appendix I |
|  | **MODIFICATIONS** |  |  |
| **10.^ǂ^** | If the intervention was modified during the course of the study, describe the changes (what, why, when, and how). | If an infant was commenced on a sedative or other medication to maintain haemostability , the intervention (NNCA model) was ceased or paused. If the sedative or other medication was ceased whilst the infant was still receiving analgesia, the intervention (return to using the NNCA model) was recommenced. | Page 6: Line 181 |
|  | **HOW WELL** |  |  |
| **11.** | Planned: If intervention adherence or fidelity was assessed, describe how and by whom, and if any strategies were used to maintain or improve fidelity, describe them. | Protocol adherence was assessed by research nurse daily by checking pain scores and adherence to algorithm. No strategies were utilised during the trial to improve adherence. This was one of the objectives of the pilot to determine if protocol adherence was possible as des cribed. ____________ | A comment was added to manuscript to make this point clearer to the reader as per below. The Principal Investigator was responsible for reviewing documented pain scores and subsequent adherence to the protocol algorithm for each 24 hour period the NNCA was being utilised. |
| **12.^ǂ^** | Actual: If intervention adherence or fidelity was assessed, describe the extent to which the intervention was delivered as planned. | One infant in the intervention group has documented pain scores as per the algorithm with subsequent appropriate use of outlined cycles as per the NNCA model. ____________ | Described in the results section page 10: line 286 |

** **Authors** - use N/A if an item is not applicable for the intervention being described. **Reviewers** – use ‘?’ if information about the element is not reported/not sufficiently reported.

† If the information is not provided in the primary paper, give details of where this information is available. This may include locations such as a published protocol or other published papers (provide citation details) or a website (provide the URL).

ǂ If completing the TIDieR checklist for a protocol, these items are not relevant to the protocol and cannot be described until the study is complete.

* We strongly recommend using this checklist in conjunction with the TIDieR guide (see *BMJ* 2014;348:g1687) which contains an explanation and elaboration for each item.

* The focus of TIDieR is on reporting details of the intervention elements (and where relevant, comparison elements) of a study. Other elements and methodological features of studies are covered by other reporting statements and checklists and have not been duplicated as part of the TIDieR checklist. When a **randomised trial** is being reported, the TIDieR checklist should be used in conjunction with the CONSORT statement (see [www.consort-statement.org](http://www.consort-statement.org)) as an extension of **Item 5 of the CONSORT 2010 Statement.** When a **clinical trial** **protocol** is being reported, the TIDieR checklist should be used in conjunction with the SPIRIT statement as an extension of **Item 11 of the SPIRIT 2013 Statement** (see [www.spirit-statement.org](http://www.spirit-statement.org)). For alternate study designs, TIDieR can be used in conjunction with the appropriate checklist for that study design (see [www.equator-network.org](http://www.equator-network.org)).
