# Supplementary material for: A Neonatal Nurse‐Controlled Model of Analgesia to Manage Post‐Operative Pain in the Surgical Neonate: A Pilot Randomised Controlled Trial
Source: J Adv Nurs. 2025 Apr 24;82(2):1725–36. doi: 10.1111/jan.16992 (PMC12810603; doi:10.1111/jan.16992)
Supplement: Supplementary file 5 — Appendix S5. [file JAN-82-1725-s004.docx]

**Appendix II Staff acceptability survey frequencies and total percentages.**

| ***Staff Acceptability of NNCA Model*** | **Scale** | n ***(%)*** |
| --- | --- | --- |
| *The NNCA model pathways are logical and able to be followed without difficulty? (n=22)* | 1  2  3  4  5 | 3 (13.6) 12 (54.5)  2 (9.1)  4 (18.2)  1 (4.5) |
| *Guidelines for use of the NNCA model are comprehensive and contain relevant and appropriate information for holistic management of pain? (n=22)* | 1  2  3  4  5 | 8 (36.4)  13 (59.1)  1 (4.5)  0  0 |
| *It is beneficial to have a formalised escalation pathway for post-operative pain management? (n=20)* | 1  2  3  4  5 | 17 (85.0)  2 (10.0)  1 (5.0)  0  0 |
| *Management of post-operative pain is simplified by having a formalised titration and weaning pathway? (n=21)* | 1  2  3  4  5 | 12( 57.1)  5 (23.8)  3 (14.3)  1 (4.8)  0 |
| *The pathways give me greater autonomy in managing infant’s comfort and pain?*  *(n=22)* | 1  2  3  4  5 | 11 (50.0)  5 (22.7)  4 (18.2)  2 (9.1)  0 |
| *The pathways improve overall analgesic management in the post-operative period? (n=22)* | 1  2  3  4  5 | 7 (31.8)  9 (40.9)  6 (27.3)  0  0 |

Staff Acceptability of NNCA Model :1( indicates strongly agree to 5 strongly disagree**),**
